# Curettage with cement augmentation of large bone defects in giant cell tumors with pathological fractures in lower-extremity long bones

**DOI:** 10.1007/s10195-016-0397-8

**Published:** 2016-02-15

**Authors:** Som. P. Gupta, Gaurav Garg

**Affiliations:** 1 Mahatma Gandhi Medical College and Hospital, Jaipur, India; 2 S.M.S. Medical College and Attached Hospitals, Jaipur, India

**Keywords:** Giant cell tumors, Curettage, Cement augmentation, Pathological fractures

## Abstract

**Background:**

Thorough curettage and cement augmentation is the procedure of choice for treating giant cell tumor lesions, particularly those associated with large defects. Its association with pathological fractures has not been studied to a great extent, although a pathological fracture following a giant cell tumor is not a contraindication to treatment by curettage and cementation. We present our experience of bone cementation following intralesional curettage for treatment of giant cell tumors of the long bones of lower limbs with associated pathological fractures.

**Materials and methods:**

A total of 38 patients who had undergone a procedure in the weight-bearing long bones of lower limbs were included in the study. The age of the patients ranged from 18−79 years with a mean age of 38.57 years. The average follow-up was 102.42 months (8.5 years) ranging from 60−186 months (5−15.5 years). Results were based on serial radiographs showing consolidation of the lesion along with a subjective clinical examination and Enneking functional evaluation noted in the patient’s records.

**Results:**

Approximately 76 % of the lesions occurred around the knee. The results were graded as excellent (72 %), good (12.82 %) fair (10.25 %) and poor (5.12 %). Four cases developed a recurrence. Apart from a few documented complications, the lesions showed good consolidation and healed well.

**Conclusion:**

Giant cell tumors of the long bones of lower limbs with an associated pathological fracture at diagnosis can be managed with thorough curettage and cement augmentation of the bone defect with a satisfactory outcome.

**Level of evidence:**

Level IV.

## Introduction

Curettage with cement augmentation involves thorough curettage of a pathological lesion from the bone and filling the residual cavity with polymethyl methacrylate (PMMA). It is particularly useful for giant cell tumors that extend to the subchondral area but do not usually invade the cartilage. Curettage and acrylic cementing for pathological fractures was first described by Wouters in 1974, followed by Persson et al. [1, 2]. Cementation using methylmethacrylate has been studied previously with good results [3], and is the procedure of choice for surgeons treating these lesions, particularly those which are associated with large defects. However, its association with pathological fractures has not been studied to a great extent, although a pathological fracture following a giant cell tumor is not a contraindication to treatment by curettage and cementation [4]. Cementation provides instant stability and sufficient quantity of filling material for the large tumor cavity. Furthermore, its exothermic property kills tumor cells and causes less recurrence. This study retrospectively evaluates our experience of bone cementation following intralesional curettage for the treatment of giant cell tumors of the long bones of lower limbs with associated pathological fractures.

## Methods and materials

We performed a retrospective analysis of clinical records, radiographs and outcomes of histologically proven cases of giant cell tumor lesions of the long bones of lower limbs with associated pathological fractures that had undergone curettage and filling of the defect with cement augmentation between 1998 and 2008. A total of 38 patients who had undergone a procedure in the weight-bearing long bones of the lower limbs were included in the study. The age of the patients ranged from 18−79 years with a mean age of 38.57 years. There were 27 males and 11 females (M:F ratio 2.4:1). The average follow-up was 102.42 months (8.5 years) ranging from 60−186 months (5−15.5 years). Inclusion criteria were histologically proven giant cell tumor lesions in the long bone of lower limbs treated with curettage and PMMA cement augmentation, with internal fixation as necessary and a minimum follow-up of 5 years. Patients with multiple lesions or tumors of the proximal fibula treated by en-bloc excision, and patients who had received adjuvant chemotherapy or radiotherapy were excluded from the study.

Treatment files were retrieved from the clinical record department. After careful history taking and a thorough physical examination, the patients were subjected to true-size antero-posterior and lateral radiographs of the pathological lesion with the adjacent joint. A chest physician was consulted to rule out pulmonary involvement for patients aged >45 years of age. A physician was also consulted if the anesthetist suspected a chest lesion during the preoperative anesthesia checkup. At the time of presentation, 15 patients had extraosseous extension, 7 had subperiosteal cortical breach, and 16 patients and cortical erosion (Table 1). Most of the cases were Campanacci grade II or III [5]. Measurements (cm) were taken of the height, width and depth of the lesion and documented. Written consent was obtained from patients before starting treatment. As a rule, pre-operative confirmation of diagnosis was performed using fine-needle aspiration cytology or open biopsy in all patients except for 11 patients who had been biopsied at another laboratory and were later referred to us for definitive management. Four cases were of local recurrence following curettage and bone grafting performed elsewhere.

**Table 1:** Details of patients presenting with giant cell tumors

Case no.	Age/sex	Site	Tumor extension	Pathological fracture	Pulmonary metastasis
1	34/M	DF	CB	P	
2*	28/F	PT	CE	P	
3	26/M	DF	CE	P	
4	22/M	DF	CE	P	
5	41/M	PT	EE	P	
6	40/F	PF	CE	P	
7	31/M	PT	EE	P	
8	33/F	DF	EE	P	
9	45/M	DT	CB	B	
10	52/M	DF	CE	P	Yes
11	18/M	PT	CB	P	
12*	47/F	DF	EE	P	
13	63/M	PT	CE	P	
14	29/F	DF	EE	P	
15	38/M	PT	EE	P	
16	39/F	PF	CB	B	Yes
17	44/M	PT	CE	P	
18	49/M	PF	CE	P	
19	41/M	DF	CE	P	
20	20/M	DF	EE	P	
21	51/M	PF	CE	P	
22	37/F	PT	EE	P	
23	23/F	DF	CE	P	
24	63/M	DT	CE	B	
25	50/F	DF	EE	P	
26	39/M	PT	CE	P	Yes
27	40/M	PF	CE	P	
28	18/M	PT	EE	P	
29*	33/M	DT	EE	P	
30	79/M	DF	EE	P	
31	54/M	PT	CE	P	
32	32/F	DF	CB	B	
33	41/M	DF	EE	P	
34	31/M	PT	CE	P	
35	36/F	PT	CB	B	
36	48/M	DF	CB	B	
37*	37/M	PF	EE	P	
38	32/M	DF	EE	P	

*PF* proximal femur, *DF* distal femur, *PT* proximal tibia, *DT* distal tibia, *CB* subperiosteal cortical breach, *CE* cortical erosion, *EE* extraosseous extension, *P* fracture seen at presentation, *B* fracture developed following biopsy

* Operated earlier with curettage and bone grafting

The surgical technique of extensive curettage was contemplated by entering either from the limiting cortex or the side of erosion, as appreciated on a radiograph and then gradually enlarging the entry to a wide cortical window that provides visualization of the entire tumor cavity and permits digital palpation of the inner tumor walls. If extension of the tumor into the soft tissues was seen, the entire pseudo-capsule was dissected circumferentially and excised completely. The intraosseous tumor bulk was scooped out completely with a large curette until smooth cortical bony surface with punctate bleeding was visible, ensuring the undersurface of window. Meticulous care was taken to ensure that all the involved bone and the possible contaminated surrounding soft tissue was excised. The curetted material was re-sent for histopathological examination. A high-speed power burr was used in all cases. Following thorough curettage, the resulting cavity was irrigated with hydrogen peroxide in all cases and phenol-dipped gauze was scraped along the cavity wall in 17 cases, followed by normal saline irrigation. The cavity was then dried and completely filled with the prepared cement mass using thumb pressure, pushing the cement into every part of the cavity. Internal fixation was used in only one patient with a lesion in the proximal femur associated with a femoral neck fracture, where titanium cannulated cancellous screws were used for fixation before cementation. Following hardening of the cement and completion of setting time, the extra cement was removed using a rongeur or an osteotome. Hemostasis was achieved and closure in layers was performed without a negative suction drain.

Plain radiographs were taken post-operatively. Appropriate antibiotics were administered and sutures were removed after 2 weeks. Range of motion exercises of the joint above and below the lesion were started after suture removal. Partial weight bearing with a pair of axillary crutches was allowed as soon as pain subsided on the third or fourth post-operative day and continued for 2 weeks. This was followed by cane support for 3–4 weeks. After a total period of 5–6 weeks, full weight bearing without support was allowed. Patients were followed up every three months for 2 years and annually thereafter with radiographs and clinical examination. Results were based on serial radiographs showing consolidation of the lesion along with a subjective clinical examination and functional outcome noted in the patient’s records. Fracture healing was assessed clinically and using plain radiographs [6]. Functional evaluation was based on the Enneking functional evaluation form [7]. Results were categorized as excellent, good, fair, or poor based on the specific numeric range assigned in the scoring system.

## Results

**Fig. 1:**
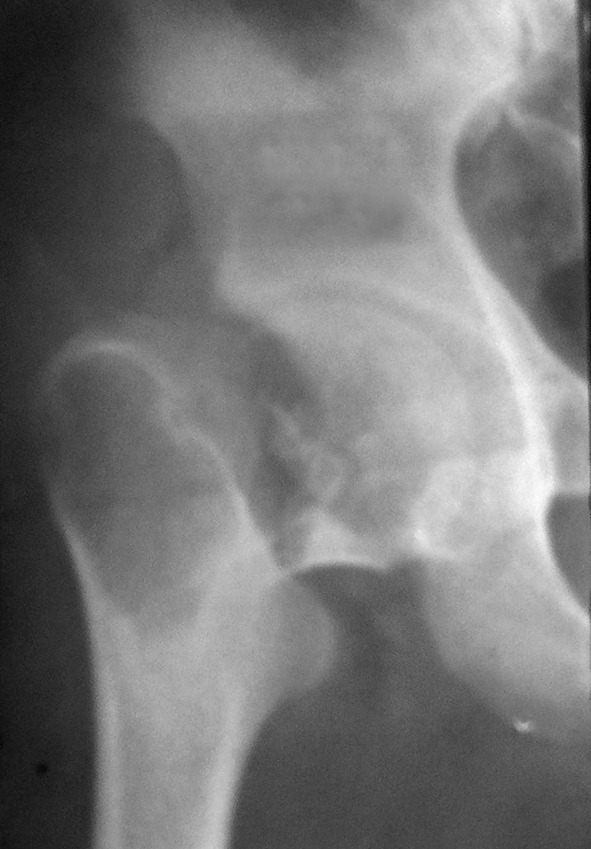
Antero-posterior radiograph showing an osteolytic lesion in the proximal femur with a pathological femoral neck fracture

**Fig. 2:**
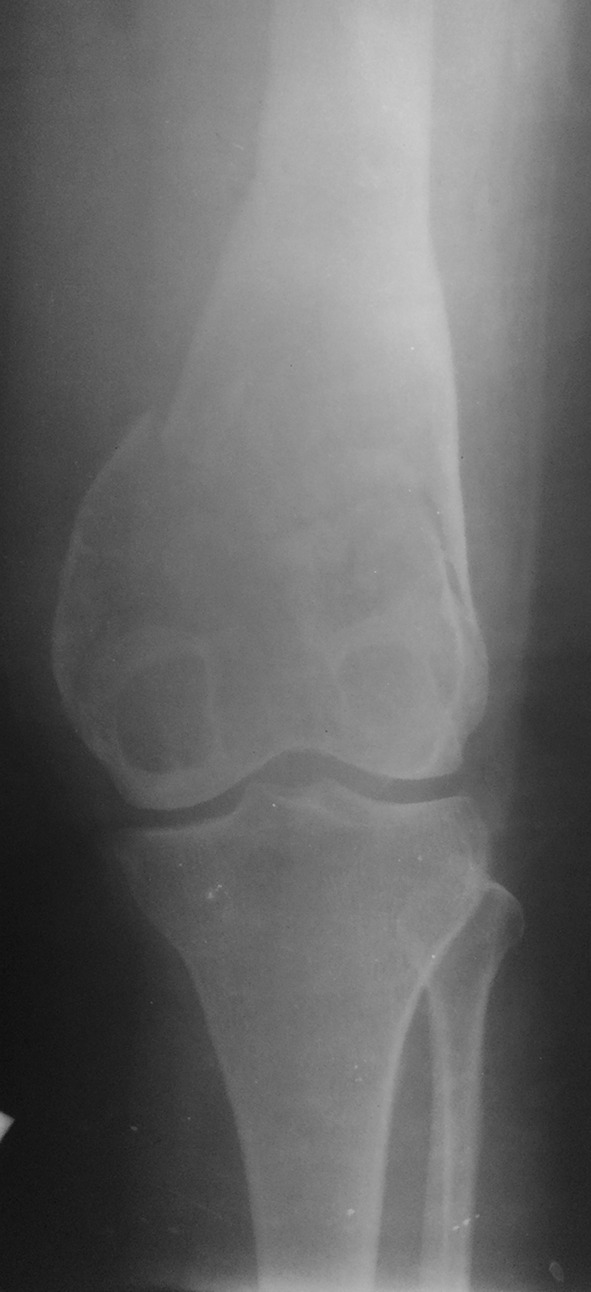
Radiograph showing an osteolytic lesion in the distal femur with an associated pathological fracture

**Fig. 3:**
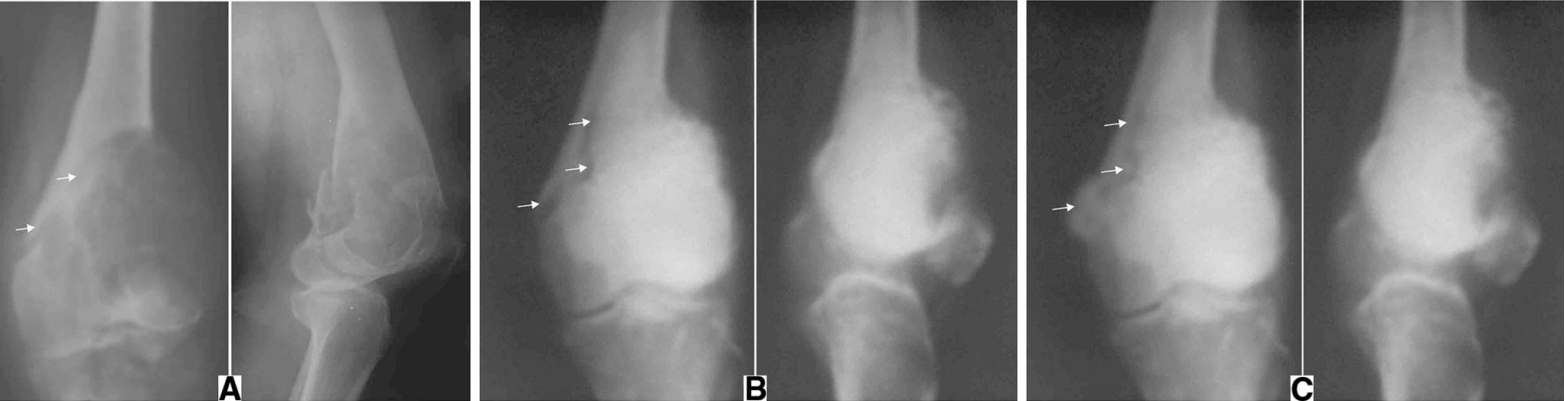
Radiographs of a 34-year-old male (case 1) showing a lesion in the distal end of the femur with a pathological fracture (**a**). Post-operative radiographs showing cementation (**b**). Sequential follow-up radiographs at 11 weeks showing good consolidation of the fracture line (**c**). (*Arrows* locating the pathological fracture)

Two-thirds of our patients were aged between 21 and 30 years. All 38 patients had lesions in the weight-bearing lower extremity long bones with an associated pathological fracture. Approximately 76 % of the lesions were reported around the knee. Various sites of lesions included proximal femur involvement in 6 patients, distal femur in 16 patients, proximal tibia in 13 patients and distal tibia in 3 patients (Figs. 1 and 2). The largest lesion measured 10 × 9 × 6 cm and smallest lesion measured 5 × 4 × 3 cm on plain radiographs. Nearly all lesions showed cortical expansion and 79 % of the lesions showed extension to the joint surface. At presentation, fractures were documented in 32 patients, and developed following biopsy in 6 patients. Two patients (cases 10 and 26) were diagnosed with pulmonary lesions at the time of presentation and one patient (case 16) developed pulmonary metastasis during the follow-up period. Among the proximal femoral lesion group, two patients reported lesions in the neck area with associated femoral neck fracture while the remaining 4 patients had lesions in the greater trochanter area with a nondisplaced fracture involving the trochanteric area. Fracture healing occurred in 33 of the 38 patients treated with curettage and cement augmentation after a mean of 16.8 weeks (range 7−39 weeks) (Fig. 3).

Three patients developed a superficial infection which was managed with prolonged antibiotic therapy. Necrosis of the overlying skin in the metadiaphyseal region of the tibia was seen in one patient and treated with repeated debridement and flap cover. Another patient treated for a lesion in the proximal femur developed a fracture of the cement mass 3 weeks post-operatively due to early weight bearing. One patient who was treated for a lesion in the distal femur developed patella-femoral arthritis in the knee joint 10 months post-operatively. The patient was managed conservatively for 6 months and was later advised to undergo total knee arthroplasty; however, the patient did not opt for surgery until the last follow-up. Four patients developed local recurrence during the follow-up period, which were clinically characterized by pain and radiologically by lysis or failure of development of the sclerotic rim between the cement and cancellous bone. In two cases, one with a lesion in the distal femur and one with a lesion in the proximal tibia, recurrence developed after 12 months and in other two cases, tibial recurrences were seen at 14 and 18 months following cement augmentation. All the recurrent lesions were of a benign nature. Magnetic resonance imaging (MRI) evaluation was performed in all four patients with local recurrences. Of these four recurrences, one patient (case 29) who was operated previously with curettage and bone grafting for a lesion in the distal tibia again showed local recurrence. All recurrences were confirmed by a second surgical histopathology examination.

**Fig. 4:**
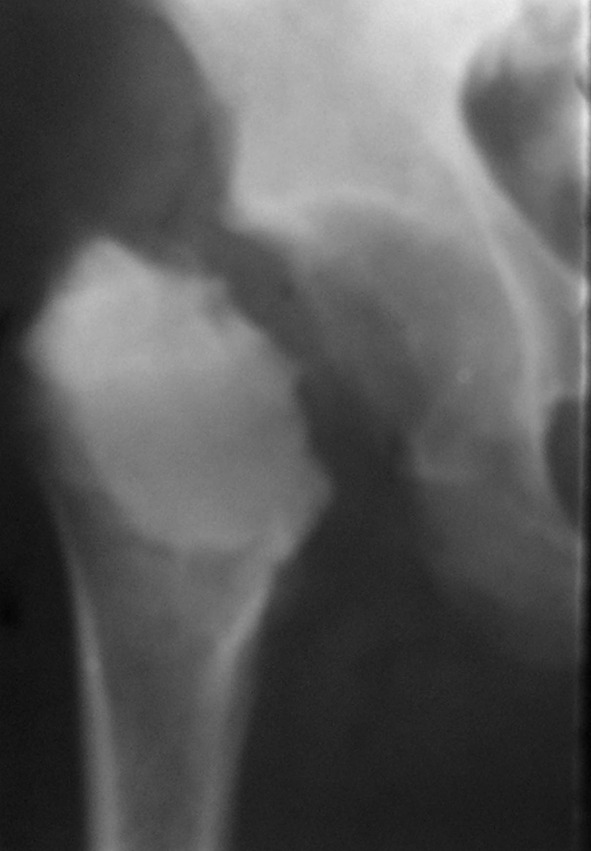
Radiograph of a 40-year-old female (case 6) showing Girdlestone resection arthroplasty with cementation seen in an osteolytic lesion

**Fig. 5:**
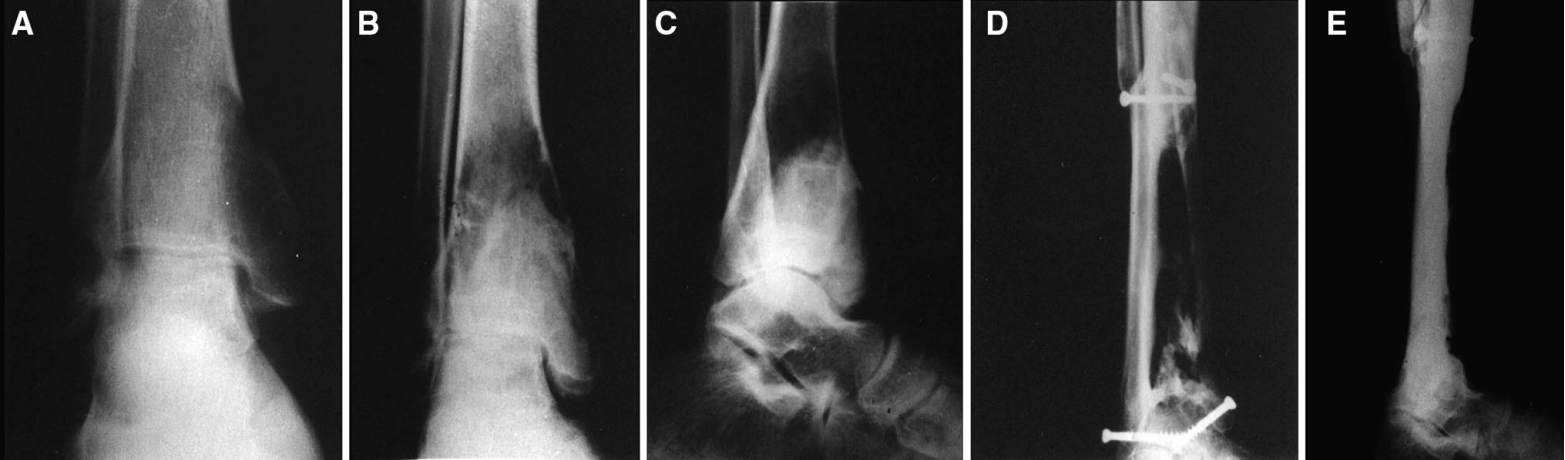
Radiograph of a 45-year-old male showing a giant cell tumor at the lower end of the tibia with a pathological fracture (**a**). Antero-posterior and lateral radiographs showing recurrence at 14 months after curettage and cementation (**b**, **c**). Follow-up radiographs at 6.5 years of the same patient treated with dual fibular grafts (**d**, **e**)

**Fig. 6:**
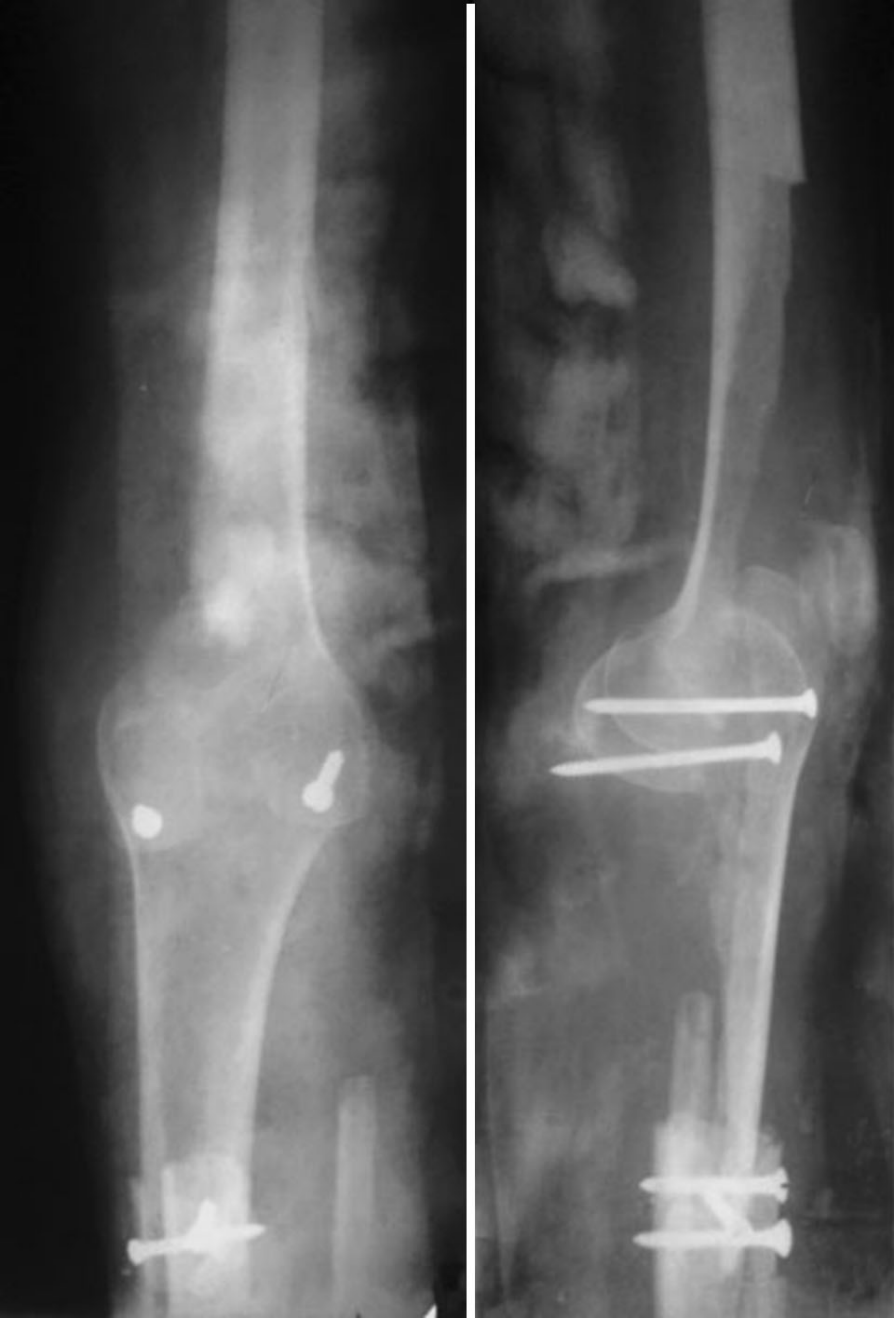
Antero-posterior and lateral radiographs of a 63-year-old male patient (case 13) showing a distal femur turnoplasty being performed for a recurrent lesion in the proximal tibia

**Fig. 7:**
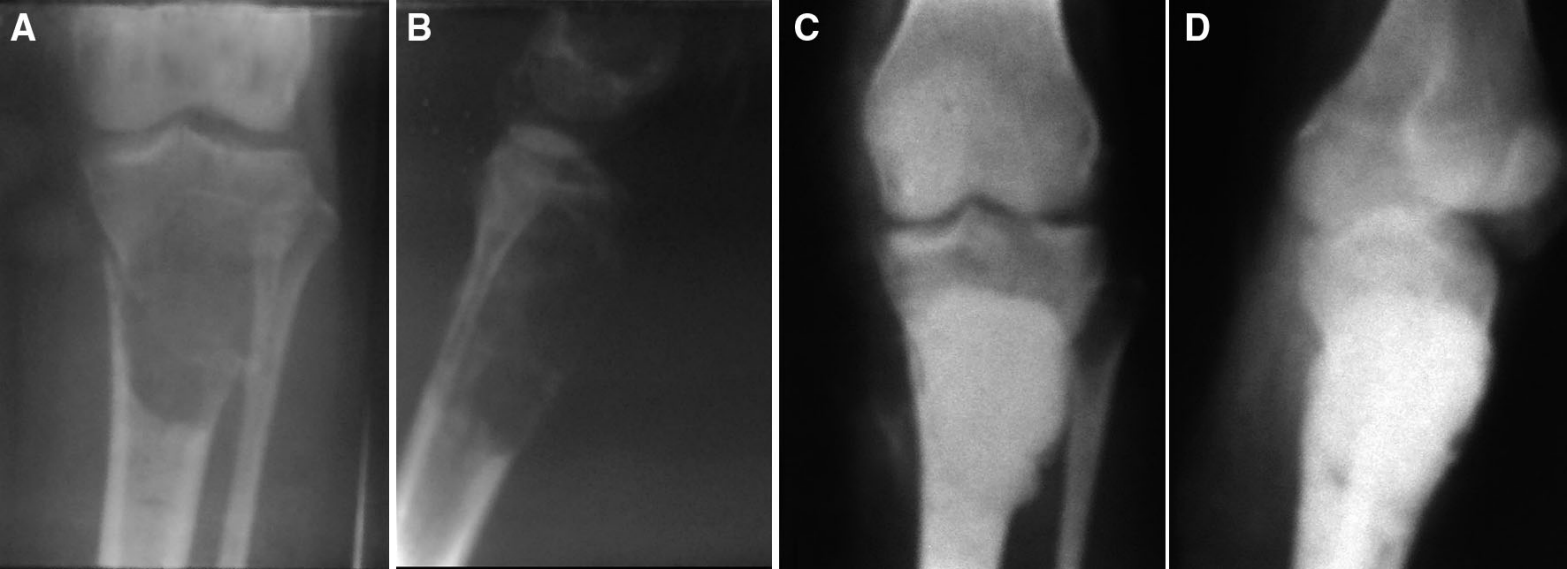
Antero-posterior and lateral radiographs of an 18-year-old male patient showing a giant cell tumor lesion in the proximal tibia with a pathological fracture (**a**, **b**). Follow-up radiographs at 14.5 years after curettage and cementation showing good consolidation of the lesion with no recurrence (**c**, **d**). The patient had normal range of motion and was asymptomatic

**Fig. 8:**
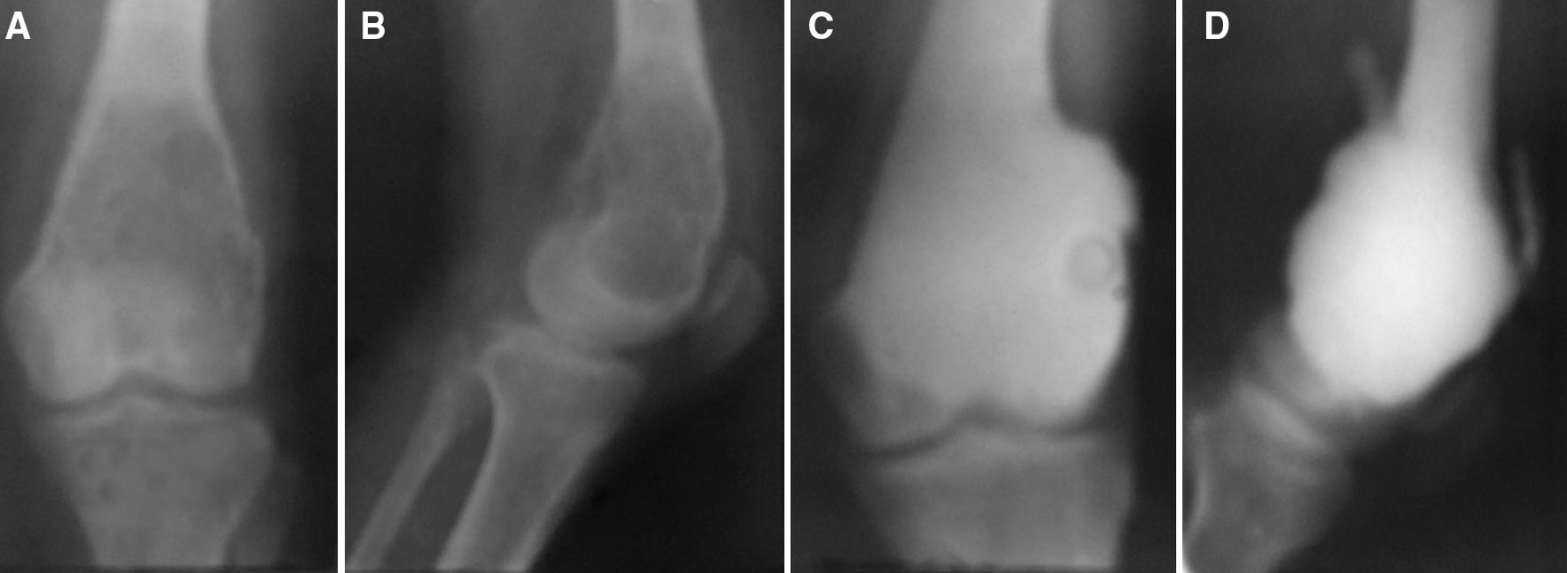
Antero-posterior and lateral radiographs of a 33-year-old female showing an extensive lesion in the distal femur with a pathological fracture (**a**, **b**). Follow-up at 8 years after after curettage and cementation (**c**, **d**). The patient had good range of motion at the knee

Alternative modes of treatment following cement augmentation were employed in four of our cases. One case of fractured cement mass in the proximal femur underwent Girdlestone resection arthroplasty (Fig. 4). Above-knee amputation was carried out in one patient with a local recurrence of the lesion 1 year following primary surgery, involving the distal femur with extensive soft-tissue involvement. One patient who was advised to undergo tumor prosthesis reconstruction could not afford to do so. In two patients who had recurrences in the distal tibia, the lower half of the tibia was excised and the limb was reconstructed using a double fibular graft in one patient (Fig. 5), and bone lengthening and ankle arthrodesis using the Illizarov technique was performed in the other patient (Fig. 6). One patient with recurrence in the proximal tibia was managed by resection of both the proximal tibia and fibula and distal femur turnoplasty (Fig. 5). Apart from these complications, the rest of the patients with a pathological fracture treated with curettage and cement augmentation healed well (Figs. 7, 8).

Using Enneking’s criteria [7] for functional evaluation, the results were graded as excellent (72 %), good (12.82 %), fair (10.25 %) and poor (5.12 %) (Table 2). One patient died during the course of follow-up due to a cause unrelated either to the lesion or surgery.

**Table 2:** Details of complications, alternative procedures being performed and final outcome

Case no.	Complications	Alternative procedures being performed	Total follow-up (months)	Results
1			112	Excellent
2			134	Excellent
3			157	Excellent
4			87	Excellent
5			63	Excellent
6	Fractured cement mass	Girdlestone arthroplasty	113	Fair
7			128	Excellent
8			96	Excellent
9	Local recurrence	Ankle arthrodesis with double fibular graft	78	Fair
10	Local recurrence	Above knee amputation	143	Poor
11			175	Excellent
12	Infection		71	Good
13	Local recurrence	Distal femur turnoplasty	93	Excellent
14			186	Excellent
15			81	Excellent
16			110	Fair
17			60	Excellent
18			91	Excellent
19	Patellofemoral arthritis		88	Fair
20			151	Excellent
21			102	Good
22			99	Excellent
23			82	Excellent
24	Infection		60	Excellent
25			78	Excellent
26			134	Good
27			90	Excellent
28			99	Excellent
29	Local recurrence, infection	Ankle arthrodesis and bone lengthening with illizarov technique	92	Poor
30			74	Excellent
31			69	Excellent
32			108	Excellent
33			94	Excellent
34			78	Excellent
35	Skin necrosis	Debridement and flap cover	119	Good
36			64	Good
37			92	Excellent
38			141	Excellent

## Discussion

While managing patients with a giant cell tumor, the surgeon must decide whether to perform an intralesional or an en bloc resection, whether to use adjuvant therapy, and what material to use to fill the resultant defect in the bone. The risk of local recurrence after en bloc resection involving the joint followed by prosthetic or allograft reconstruction is lower than after an intralesional procedure [6, 8, 9]. However, the risk of long-term complications makes this treatment generally inappropriate for giant cell tumors of bone [7–10].

Extended curettage is the commonest modality of treatment for giant cell tumors but the residual large bony defect following curettage is a major concern for the treating surgeon. Small defects can be left alone and the cavities fill up with blood which coagulates to form a clot which later becomes ossified and forms bone [11, 12]. Large defects should be filled with bone graft or substitutes such as cement, hydroxyapatite, or tricalcium phosphate [13]. This subsequently led to the evolution of intralesional curettage followed by packing of the defect with methyl methacrylate cement, which was first described in 1969 by Vidal et al. [14].

Curettage and acrylic cementing for pathological fractures has been described previously [15]. Wuisman et al. [16] treated a pathological fracture of the proximal humerus by curettage and cement augmentation but the cement was later removed and autogenous bone chips were inserted. Pals and Wilkins [17] reported good results with no recurrences in 5 patients with pathological fractures treated by open reduction and cementing followed by application of allograft bone chips to the hardened cement. Studies have demonstrated the efficiency of bone substitutes like calcium phosphate as a filling agent and sufficient evidence exists to support the fact that the joint function is not compromised with time even after the use of subchondral cement [18–20]. Healing of pathological fractures through the femoral neck is a difficult scenario, as we report only one patient (case 6) with a fair outcome. Furthermore, cement is not a biological material and it is strong in compression but relatively weak when subjected to shear and torsion. Hence, its use in lesions involving the head and neck of the femur may result in an increased chance of fractures through cement.

PMMA provides immediate stability that allows early restoration of joint motion and weight bearing, and also facilitates recognition of radiological contrast with the surrounding bone and detection of any lytic recurrent zone. As no stainless steel implant was used to support the fractures in this study, MRI evaluation can be performed to detect recurrences. Should recurrence occur, this method will not compromise other surgical alternatives. Moreover, its exothermic property kills tumor cells and causes less recurrence [3, 21]. If suspicious zones are identified, they must be curetted and the material examined histologically. The extra cavity may then be filled with additional cement, without disturbing the main mass of cement previously introduced. The possible disadvantages of cement augmentation include infection, premature osteoarthritic changes of the adjacent joint and chronic effusion in the joint. It may influence the rate of bone remodeling by affecting bone metabolism and trabeculae may be weakened by changes in the mechanical environment. The experience to date with PMMA has been extremely promising with very low rates of local recurrence.

The recurrence of giant cell tumors of bone after surgery is statistically to be expected within the first 2 years although late recurrences are known. Backley et al. concluded in their study that the risk of local recurrence after curettage with high-speed burr and reconstruction of the defect with an autogenous graft or allograft bone is similar to that observed after use with cement and other adjuvant treatments [22]. In two larger studies, five of seventeen patients and eight of nineteen patients had recurrence after management with cement; these rates are equivalent to those reported after treatment without cement [23, 24]. The ability of PMMA to control giant cell tumors and minimize the chances of local recurrence has been confirmed repeatedly. The addition of phenol and hydrogen peroxide as an adjuvant may further reduce the chance of local recurrence. The success of local adjuvants, especially PMMA suggests that wide en bloc excision is no longer necessary and should not be recommended in the primary management of giant cell tumors of bone.

Some authors have recommended subsequent removal of cement followed by autogenous bone grafting. However, some authors think this would be difficult and could result in further damage to surrounding bone, including subchondral and articular surfaces [25]. None of the cases in the present series showed degenerative changes in the knee joint except for one patient with patella-femoral arthritis; however, the cavities were appreciably large and extended to the subchondral bone. The absence of degenerative changes in such cases indicates that the fear of subsequent osteoarthritis is largely unfounded.

Pathological fractures are not a contraindication for curettage and cement augmentation and, on the contrary, offer early weight-bearing mobilization supported with a functional brace. This promotes healing of the fracture contrary to the belief that surgery will disseminate the tumor cells into the soft tissues and adjacent joint [26]. Our study had some limitations. As we only included cases treated previously with this particular method, it is a subjective rather than an objective outcome. Whether this procedure should be performed immediately or can be postponed until fracture healing cannot be deduced from this study.

This retrospective study allows us to conclude that giant cell tumors of the long bones of lower limbs with associated pathological fractures at diagnosis can be managed with thorough curettage and cement augmentation of the bone defect with a satisfactory outcome.
